# Characterization of Carbapenem-Resistant Acinetobacter baumannii Isolates from Clinical Specimens

**DOI:** 10.1128/MRA.00571-21

**Published:** 2021-07-22

**Authors:** Khansaa Abdullah, Peter C. Iwen, Baha Abdalhamid

**Affiliations:** a Department of Pathology and Microbiology, University of Nebraska Medical Center, Omaha, Nebraska, USA; University of Maryland School of Medicine

## Abstract

Carbapenem-resistant Acinetobacter baumannii is an urgent threat worldwide. This bacterium is associated with high morbidity and mortality, with limited available treatment options. Here, we report the draft genome sequences of five carbapenem-resistant Acinetobacter baumannii isolates from human samples.

## ANNOUNCEMENT

Carbapenem-resistant Acinetobacter baumannii causes septicemia, wound infections, pneumonia, and urinary tract infections and is a serious threat, especially to hospitalized patients (1, 2). This article reports on five draft genome sequences of carbapenem-resistant Acinetobacter baumannii isolates collected from clinical specimens. This study did not require institutional review board (IRB) approval since it was performed on bacterial isolates that did not include patient protected health information.

Specimens from human wounds were cultured on blood agar, MacConkey agar, and chocolate agar plates and incubated overnight at 5% CO_2_ and 37°C (3). For organism identification and susceptibility, the MicroScan WalkAway system with the Gram-negative conventional identification and antibiotic susceptibility testing (ID/AST) panel (Beckman Coulter, CA) was used as recommended by the manufacturer. Acinetobacter baumannii isolates resistant to both imipenem and meropenem were subsequently sequenced. After overnight culture on blood agar plates at 5% CO_2_ and 37°C, genomic DNA from all isolates was extracted using a MagNA Pure compact nucleic acid isolation kit I using a MagNA Pure compact instrument (Roche Diagnostics, IN, USA). The DNA concentration and quality were detected using a NanoDrop 2000 UV-visible (UV-Vis) spectrophotometer (Thermo Fisher, MA, USA) and a Qubit 3.0 fluorometer (Invitrogen, CA, USA), respectively. The Nextera XT library prep kit (Illumina, CA, USA) was used to construct bacterial genomic DNA libraries. Draft genome sequencing was performed using the MiSeq platform (Illumina) with 150-bp paired-end chemistry. The Phred quality score (QS30 > 75%), percentage of clusters passing filters (>80%), and cluster density (600 to 1,300) were the parameters used to assess the quality of the run. For the bioinformatics pipeline, default parameters were used for all software unless otherwise specified. FastQC 0.11, Trimmomatic 0.33, SPAdes 3.15, and BBMap 38.84 were used for FASTQ quality reads, trimming, *de novo* assembly, and fasta filtration, respectively (4–7). Quast 4.1 and the NCBI Prokaryotic Genome Annotation Pipeline (PGAP) 4.8 were used for quality checking of the assembly and genome annotation (8, 9). The Abricate 1.0 CARD database was used for carbapenem-resistant gene detection, and MLST 2.0 was used for multilocus sequence typing (10–12). 

These study data provide the genetic basis to determine the antimicrobial resistance, pathogenesis, and phylogenetic relatedness of these strains.

### Data availability.

These genomic sequences are available at NCBI GenBank under BioProject accession number PRJNA673907. The strain characteristics and accession numbers are provided in Table 1.

**TABLE 1:** Summary characteristics of whole-genome sequencing for carbapenem-resistant Acinetobacter baumannii strains

Characteristic^*a*^	Data for strain:				
	B185	B230	B235	B238	NPHL200664
No. of reads	621,383	1,329,559	945,603	544,582	610,473
No. of contigs	191	125	186	163	278
Largest contig length (bp)	140,621	364,787	364,906	224,253	160,086
Total length (bp)	3,944,773	3,983,987	3,985,827	3,993,946	3,929,735
GC content (%)	39.02	39.01	39.01	39.03	39.06
*N*_50_ (bp)	47,861	153,760	131,455	90,002	37,380
MLST	2	2	2	2	2
Carbapenemase	OXA-83	OXA-109	OXA-109	OXA-109	OXA-66
BioSample accession no.	SAMN16629664	SAMN16629665	SAMN16629667	SAMN16629668	SAMN16644270
SRA accession no.	SRR12981437	SRR12981436	SRR12981435	SRR12981434	SRR12981433
GenBank accession no.	JADKYG000000000	JADKYF000000000	JADKYE000000000	JADKYD000000000	JADIIJ000000000

a MLST, multilocus sequencing type; SRA, Sequence Read Archive.
